# Sellar Region Lesions and Intracranial Aneurysms in the Era of Endoscopic Endonasal Approach

**DOI:** 10.3389/fendo.2021.802426

**Published:** 2021-01-04

**Authors:** Siyu Yan, Yifan Liu, Chang Liu, Li Yang, Yun Qin, Ran Liu, Shan Wang, Xue Li, Wenjie Yang, Lu Ma, Chao You, Liangxue Zhou, Rui Tian

**Affiliations:** ^1^ Department of Neurosurgery, West China Hospital, Sichuan University, Chengdu, China; ^2^ West China School of Medicine, West China Hospital, Sichuan University, Chengdu, China; ^3^ Department of Radiology, West China Hospital, Sichuan University, Chengdu, China; ^4^ 4Engineering Research Center of Medical Information Technology, Ministry of Education, West China Hospital, Sichuan University, Chengdu, China; ^5^ Department of Clinical Research Management, West China Hospital, Sichuan University, Chengdu, China

**Keywords:** intracranial aneurysm, computed tomography angiography, endoscopic endonasal transsphenoidal surgery, sellar mass lesions, strategy

## Abstract

In the clinical practice of neurosurgery, the endoscopic endonasal approach (EEA) has been the mainstream approach in the management of sellar region diseases. However, clinicians have come to realize that EEA procedure is associated with intraoperative hemorrhage. Due to the limited surgical field and poor proximal control under endoscope, massive hemorrhage always leads to severe complication or even perioperative death. Previously, intraoperative hemorrhage used to be attributed to endoscopic intervention of cavernous sinus or internal carotid artery, but our recent understanding of EEA indicated that preoperatively complicated intracranial aneurysms (IAs) may play a role. In this article, we retrospectively reviewed the baseline characteristics, treatment strategy, pathology, intraoperative findings, as well as radiological profiles of sellar region lesions complicated with IAs. With the focus put on the high comorbidity rate of sellar region lesions and IAs, we did further statistical analysis to sketch the outline of this coexisting circumstance and to emphasize the importance of computed tomography angiography (CTA) as routine EEA preoperative examination. Thorough patient-surgeon communication should be proceeded before the formulation of an individualized treatment strategy.

## Introduction

Sellar region lesions are a group of rare diseases in the clinical practice of neurosurgery, with various pathological classifications including pituitary adenomas, craniopharyngioma, Rathke’s cyst, meningioma, pituitary abscess, glioma, and so on. Patients usually presented with endocrinological dysfunctions and neurological manifestations such as headache, oculomotor nerve palsy, blurred vision, or even visual loss. Thus, primary diagnosis should be made based on endocrinological test and enhanced sellar magnetic resonance imaging (MRI), with or without visual field examination. Once confirmed, surgical resection should be the first-line treatment for most of the circumstances (1).

As for surgical modalities of sellar region lesions, there has been an evolution corridor in the history of neurosurgery, from open surgery to microscopic transoral surgery, then from microscopic transnasal transsphenoidal surgery to the endoscopic endonasal approach (EEA) (2–5). Until today, EEA has become an established treatment for sellar region lesions by the benefit of its minimal invasiveness and improved surgical field (6, 7). The common procedures involve pulling and dragging movements during the EEA could cause surgical damage of parasellar region structures and sometimes even result in massive hemorrhage, which is thought to be the most severe intraoperative complication. It is generally acknowledged that such an emergency is attributed to the damage of parasellar structures such as the internal carotid artery (ICA) or cavernous sinus in EEA (8). However, our recent understanding of the modality indicated that preoperatively complicated intracranial aneurysms (IAs) may also play a role with the stability of the IAs may be affected, as well as parasellar region around it, and the IAs which locate on the circle of Willis could in a greater risk.

According to literature, the prevalence of IAs can be as high as 3.2% (9), and the prevalence of sellar region lesions ranges from 10% to 20% among the general population. Thus, the co-existence of IAs and sellar region lesions used to be taken as a rare condition by inertial thinking and drawn little attention during preoperative evaluation. In the recent decades, a few studies focused on this coexisting circumstance and reported the prevalence varied between 0.9% and 8.3% (10–14),while the real-world prevalence data of sellar region lesions complicated with IAs among Chinese population is limited. Also, the therapy challenges of complicated occasional IAs in the era of EEA modality for sellar region lesions has not been well discussed. In this article, we presented a retrospective observational study to sketch the clinical and radiological characteristics of patients diagnosed with sellar region lesions co-existed with IAs, with the emphasis on the discussion of preoperative evaluation and individualized formulation of treatment strategy under the high comorbidity rate.

## Materials and Methods

### Study Population

Continuous patients diagnosed with sellar region lesions from December 2016 to November 2020, in the Department of Neurosurgery, Shangjin branch of West China Hospital, Sichuan University, were retrospectively screened. On the basis of the electronic health records, the detailed inclusion criteria were as follows: (1) age ≥ 18 years; (2) sellar region lesions diagnosed with the composite of clinical presentations, laboratory tests, histological investigations, and enhanced sellar magnetic resonance imaging (MRI); (3) complicated with IAs diagnosed by computed tomography angiography (CTA), digital subtraction angiography (DSA), or intraoperative findings; (4) be capable to offer informed consent independently or with the help of relatives. While exclusion criteria were as follows: (1) patients with other systemic diseases contradict surgical intervention; (2) patients lack of valid radiological evidence of IA diagnosis.

### Data Acquisition

This study was consistent with the 1964 Declaration of Helsinki and its later amendments or comparable ethical standards, and was reviewed and approved by the Institutional Review Board and Ethics Committee of West China Hospital, Sichuan University. Written informed consent to participate in this study was provided by the participants’ legal guardian/next of kin.

Baseline characteristics including sex, age, tobacco use, alcohol intake, history of hypertension, history of subarachnoid hemorrhage (SAH), onset symptoms, preoperative and postoperative hormone load, and treatment strategies were collected from electronic health records of all included patients.

Based on the histological evidence or evaluated comprehensively by the laboratory results of endocrinology examination before surgeries and clinical symptoms, tumors of the sellar region were classified according to the 4^th^ edition of the World Health Organization (WHO) classification of endocrine tumors (15, 16), among which including corticotroph adenoma, somatotroph adenoma, lactotroph adenoma, thyrotroph adenoma, gonadotroph adenoma, null-cell adenoma, craniopharyngioma, and chordoma. On the grounds of the preoperative enhanced sellar MRI, the invasiveness of cavernous sinus was evaluated and graded in line with the Knosp classification (17) independently by two neurosurgeons on the Picture Archiving and Communication System. Sellar region tumors graded from 0 to 2 were defined as noninvasive while from 3 to 4 were invasive ones. Elevation of the optic nerve was investigated as well.

Features of intracranial IAs were also reviewed by two neurosurgeons independently that relevant parameters were extracted from the outcome of multiplanar 3D reconstruction on the Picture Archiving and Communication System. IA was defined as an abnormal outpouching in the wall of cerebral blood vessels (18). The suspicious localized dilation judged by both reviewers was excluded. The number, location, size, neck width, and direction of detected IAs of patients were collected. The location of IAs was recognized as ICA and its segments in accordance with the Bouthillier classification (19), among which includes anterior cerebral artery (ACA), middle cerebral artery (MCA), posterior cerebral artery (PCA), anterior communicating artery (AoCA), and posterior communicating artery (PoCA). According to measured neck width, the dome-to-neck ratio was calculated for each scanned IAs, with the relatively high or low ruptures risks respectively during the EEA, then it would be divided into wide-neck (dome-to-neck ratio ≥ 2 or neck width < 4 mm) or narrow-neck (dome-to-neck ratio < 2 or neck width ≥ 4 mm) (20). At the same time, IAs were classified based on their direction to the cavernous sinus as well, which includes point to, in parallel with, or deviate from the cavernous sinus, presenting the fairly high, moderate, or mild rupture risks under the limited surgical field.

The prognosis was indicated by information compounding survival state, any newly emerging abnormalities, as well as any improvement of symptoms before the surgery collected by telephone follow-up.

### Statistical Analysis

A flowchart was applied to show the process of participants selection. SPSS software version 26.0 (IBM Corp., Armonk, New York, USA) was used to analyze the collected data in this study. Categorical variables were described with numbers and percentages, while continuous variables were described with means ± standard deviation.

## Results

**Figure 1:**
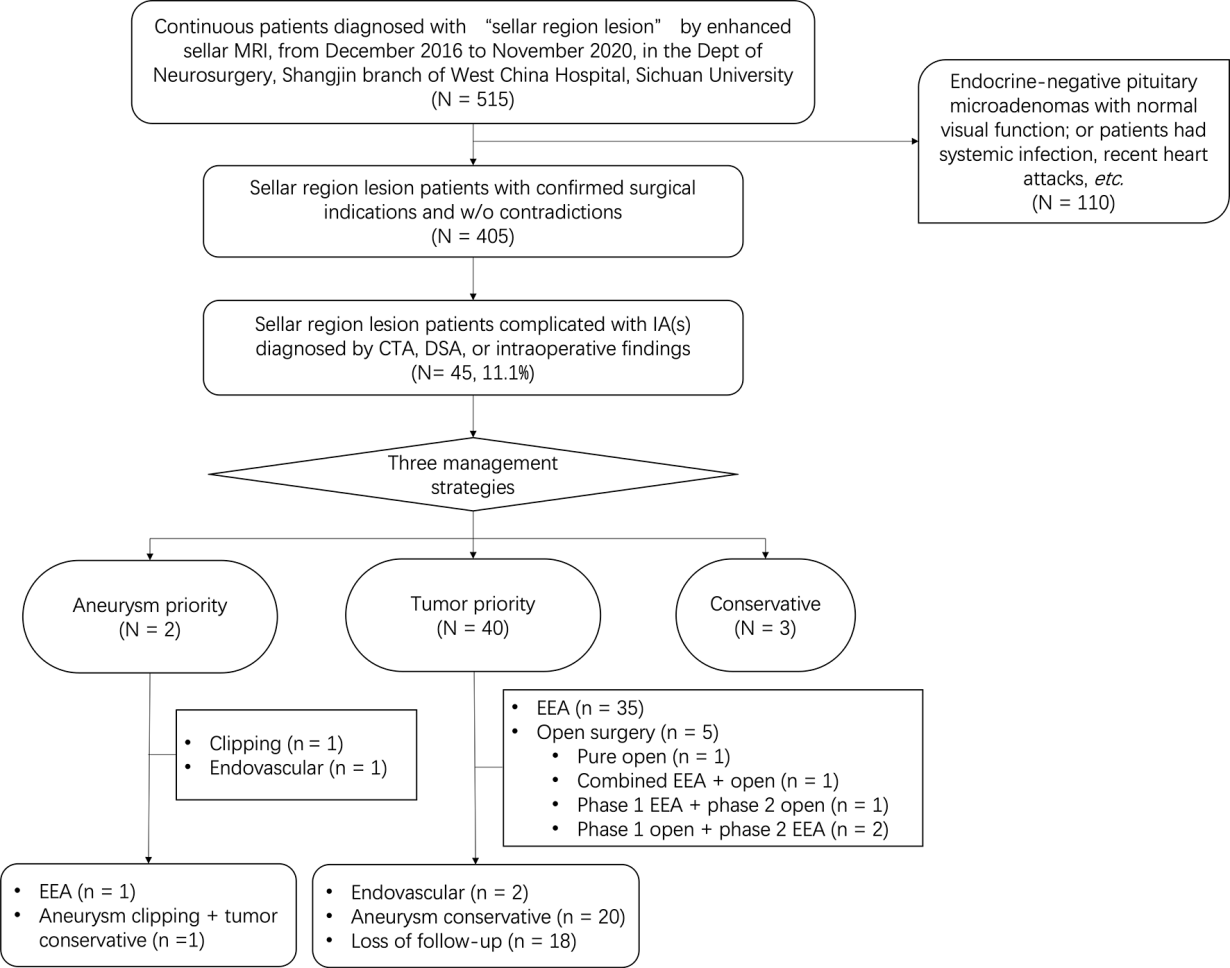
Patient recruitment flowchart of sellar region lesions complicated with IAs.

**Figure 2:**
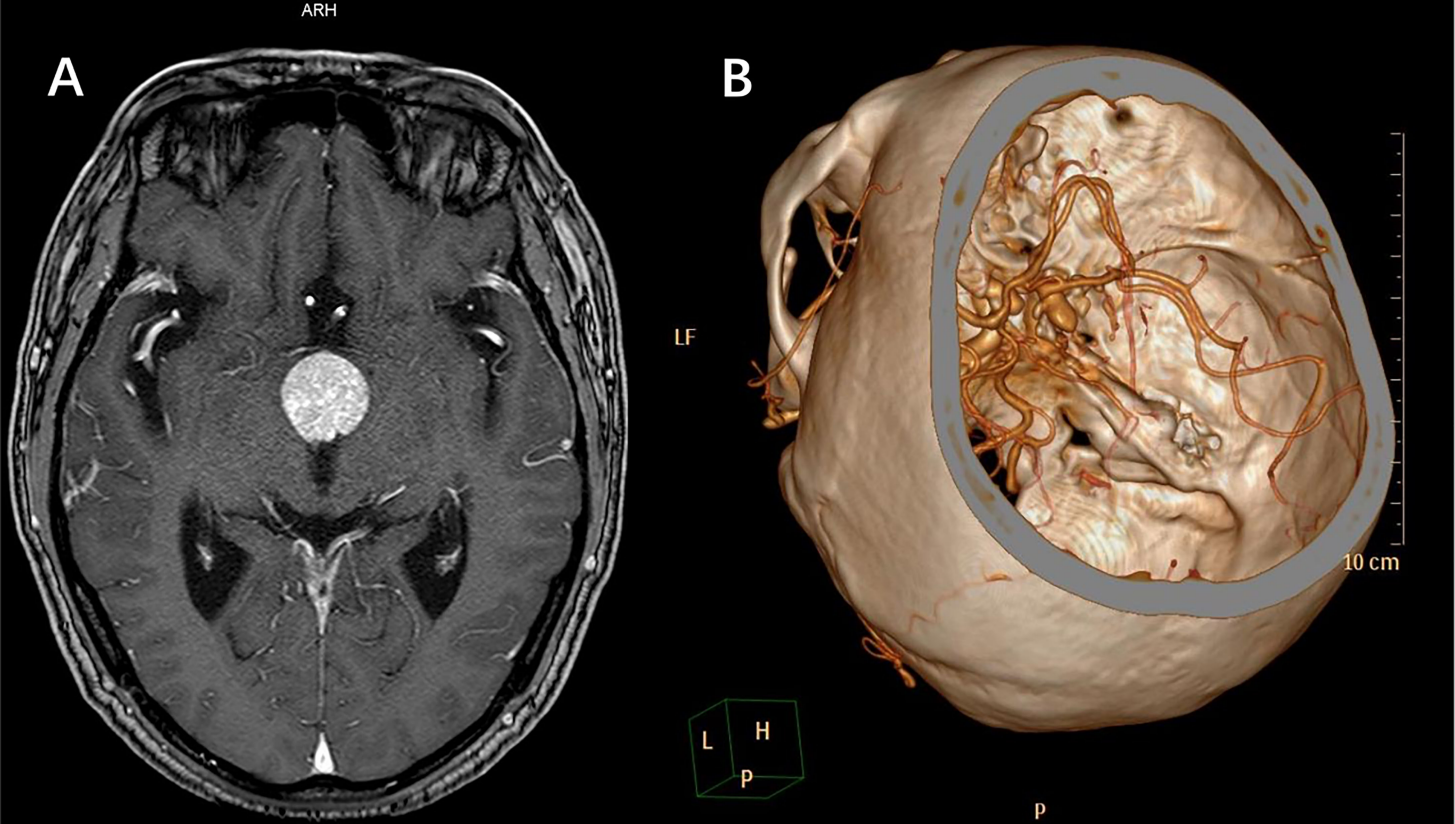
A case of typical radiological profile of sellar region lesion complicated with IAs. **(A)** MRI indicated sellar region lesion. **(B)** Further CTA revealed a partially thrombotic pseudoaneurysm formed from ruptured IA, and the IA located close to the sellar region lesion. In such occasion, incomplete preoperative examinations without CTA can lead to intraoperative massive hemorrhage, even perioperative death.

A total of 515 continuous patients diagnosed with “sellar mass” by enhanced sellar MRI were reviewed through medical records. 110 of them were either endocrine-negative pituitary microadenomas with normal visual function or had surgical contradictions (such as systemic infection and recent heart attacks), 405 patients were further evaluated. By further review of radiological and intraoperative findings, we included 45 patients complicated with IAs diagnosed by CTA, DSA, or intraoperative findings. Patient recruitment flowchart was shown in **Figure 1**. The baseline information of included patients was shown in **Table 1**. The typical radiological profile of a sellar mass patient complicated IAs is shown in **Figure 2**.

**Table 1:** Baseline characteristics of included patients.

Characteristic	n (%), mean ± SD, or description
Sex(M/F)	24/21
Age (years)	56.38 ± 11.21
Tobacco use	13/45 (28.9%)
Alcohol intake	9/45 (20.0%)
History of hypertension	16/45 (35.6%)
History of SAH	0/45 (0.0%)
Onset symptoms	
Dizziness or headache	26/45 (57.8%)
Acute visual loss	23/45 (51.1%)
Oculomotor nerve palsy	3/45 (6.7%)
Endocrinological symptoms	4/45 (8.9%)
Other symptoms	Nausea, vomiting, weakness or numbness of limbs, unilateral facial pain, and sexual dysfunction
Treatment strategies	
Underwent surgeries for tumor first and follow-up for IAs	40/45 (88.9%)
EEA	35/40 (87.5%)
Craniotomy	5/40 (12.5%)
Total tumor excision	21/40 (52.5%)
Subtotal tumor excision	19/40 (47.5%)
Underwent surgeries for IAs first	2/45 (4.4%)
Underwent surgeries for both tumor and IAs	0/45 (0.0%)
Conservative w/o any surgeries during follow-up	3/45 (6.7%)

NOTES: SAH, subarachnoid hemorrhage; IAs, unruptured intracranial aneurysms; EEA, endoscopic endonasal approach.

Clinical characteristics of all the 45 included sellar region lesions were listed in **Table 2**, including histological and endocrinological subtype and tumor invasiveness. Thus, 38 out of 45 (84.4%) patients proved to be pituitary adenoma, and 34 out of 45 (75.6%) patients had noninvasive tumors (Knosp classification 0-2). The operation information among patients presenting different tumor invasiveness was shown in **Table 2-S1**, while the preoperative and postoperative hormone loads in different subtypes of functional pituitary adenomas was presented in **Table 2-S2**.

**Table 2:** Clinical characteristics of sellar region lesions.

Characteristic	n (%) or mean ± SD
Histological and endocrinological subtype	
Pituitary adenoma	38/45 (84.4%)
Corticotroph adenoma	1/38 (2.6%)
Somatotroph adenoma	2/38 (5.3%)
Lactotroph adenoma	10/38 (26.3%)
Thyrotroph adenoma	1/38 (2.6%)
Gonadotroph adenoma	1/38 (2.6%)
Null-cell adenoma	23/38 (60.5%)
Craniopharyngioma	3/45 (6.7%)
Chordoma	4/45 (8.9%)
Tumor invasiveness	
Noninvasive tumors (Knosp classification 0-2)	34/45 (75.6%)
Invasive tumors (Knosp classification 3-4)	11/45 (24.4%)
Optic nerve elevation	34/45 (75.6%)

Clinical characteristics and radiological profiles of IAs of all the 45 included patients was shown in **Table 3**. Detailed information of IAs included number, location, size (maximal diameter), dome to neck ratio, as well as pointing direction. Thus, 35 out of 45 (77.8%) patients had only single aneurysm, 19 out of 58 (32.8%) IAs located on ICA C6 segment (ophthalmic segment), 30 out of 58 (51.7%) IAs were micro-aneurysms with diameter less than 3 mm, 39 out of 58 (67.2%) IAs were narrow-neck (dome to neck ratio < 2). As for the pointing direction of IAs, 23 out of 58 IAs (39.7%) pointed to the cavernous sinus, and 21 out of 58 (36.2%) IAs pointed deviated from the cavernous sinus or far away. And the operation information among patients with different IAs size, different dome-to-neck ratio of IAs, as well as different direction of IAs, were shown in **Tables 3-S1**–**3-S3**, respectively.

**Table 3:** Clinical characteristics and radiological profiles of IAs.

Characteristic	n (%) or mean ± SD
Number	58
1	35/45 (77.8%)
2	7/45 (15.5%)
3	3/45 (6.7%)
Location	
ICA C1 (cervical segment)	1/58 (1.7%)
ICA C2 (petrous segment)	0 (0.0%)
ICA C3 (lacerum segment)	0 (0.0%)
ICA C4 (cavernous segment)	4/58 (6.9%)
ICA C5 (clinoid segment)	7/58 (12.1%)
I CA C6 (ophthalmic segment)	19/58 (32.8%)
I CA C7 (communicating segment)	14/58 (24.1%)
ACA	1/58 (1.7%)
MCA	4/58 (6.9%)
PCA	1/58 (1.7%)
AoCA	0/58 (0.0%)
PoCA	5/58 (8.6%)
Others	VA (1/58, 1.7%); ICA, segment unidentified (1/58, 1.7%)
Size (Maximal diameter)	
d≥10mm	3/58 (5.2%)
3mm≤d<10mm	22/58 (37.9%)
d<3mm	30/58 (51.7%)
Without description	3/58 (5.2%)
Dome to neck ratio	
Wide-neck(ratio≥2)	16/58 (27.6%)
Narrow-neck(ratio<2)	39/58 (67.2%)
Without description	3/58 (5.2%)
Pointing direction	
Point to the cavernous sinus	23/58 (39.7%)
In parallel with the cavernous sinus	8/58 (13.8%)
Deviate from the cavernous sinus or far away	21/58 (36.2%)
Cannot be identified	6/58 (10.3%)

NOTES: ICA, internal carotid artery; ACA, anterior cerebral artery; MCA, middle cerebral artery; PCA, posterior cerebral artery; AoCA, anterior communicating cerebral artery; PoCA, posterior communicating cerebral artery; VA, vertebral artery.

As for follow-up data of long-term prognosis, detailed information was presented in **Table 4**. Follow-up information included follow-up rate, survival state, newly emerging abnormalities, as well as improvement of symptoms. A total of 27 out of 45 (60%) patients were on follow-up, and 21 out of 27 (77.8%) patients reached complete improvement.

**Table 4:** Prognosis of included patients *via* follow-up.

Characteristics	n (%) or description
Follow-up rate	27/45 (60.0%)
Survival state	
Survival	27/27 (100.0%)
Disabled	3/27 (11.1%)
Dead	0/27 (0.0%)
Newly emerging abnormalities	3/27 (11.1%, including loss of vision, speech disorder, and paralysis)
Improvement of symptoms	
Complete improvement	21/27 (77.8%)
Partial improvement	6/27 (22.2%)
No improvement	0/27 (0.0%)
Impaired improvement	0/27 (0.0%)

## Discussions

In this article, we retrospectively reviewed the baseline characteristics, treatment strategy, pathology, intraoperative findings, as well as radiological profiles of sellar region lesions complicated with IAs. Among 45 included patients (M/F, 24/21), the mean age is 56.38 ± 11.21 ( ± SD) years old. Dizziness, headache, and acute visual loss are the most common symptoms of onset, suggesting that atypical manifestations such as dizziness and headache should not be ignored in clinic practice.

Importantly, we found that the real-world comorbidity rate of sellar region lesions and IAs in the Chinese population is greater than the traditionally perceived data reported from 1978 to 2016 by foreign literature, which varied from 0.9% to 6.0% (10–13), as well as greater than that reported by another Chinese neurosurgical team (8.3%) (14). However, in our study, the overall prevalence of sellar region lesions co-existed with the IAs is as high as 11.1%. The difference can be attributed to the rapid development of radiological techniques. More advanced medical imaging equipment brings an increased detection rate compared to the autopsy-confirmed times in history (21, 22). By referring to the data distribution of the baseline demographic data, tumor biology information, and radiological features of IAs, our reported data has been in accordance with clinical reality and proved reliable. Therefore, the 11.1% high prevalence of comorbidity brings out new challenges to neurosurgeons that cerebrovascular screening for occasional IAs should be taken into considerations before the surgery. Rupture of undetected IAs during EEA procedures can be catastrophic and always leads to patient death on operation table. CTA is a non-invasive imaging modality with greater availability and lower cost to detect IAs (23), which may benefit EEA patients for sellar region lesions. To decrease the risk of unprepared intraoperative IAs rupture during an EEA operation, which is impossible to achieve proximal control of ICA, we strongly suggested that CTA be written into related clinical guidelines as routine preoperative evaluation before EEA.

When there is known IAs complicated with sellar region lesions, the IAs circumstances must be treated with super cautiousness and individualized strategy of treatment must be formulated for safety concerns. By the statistical sketching of outline of this coexisting circumstance, we found the major pathological classifications of patients were pituitary adenoma (84.4%), and more than half (60.5%) were null-cell adenoma, who barely have endocrinological discomforts and usually present with compression symptoms. So, the null-cell adenomas are usually moderate to huge in size, and tender to be invasive with greater Knosp classification. In this way, there might be more pulling movements with a higher risk of intraoperative interruption of the complicated IAs during EEA. The direction of the IAs also contributes to a certain risk of IAs rupture for their different accessibilities during the separation. **Table 3-S3** demonstrated that nearly two-thirds of IAs do not deviate from the cavernous sinus. These IAs are at greater risk of rupture during EEA operation.

An individualized treatment strategy needs to be formulated for patients who have sellar region lesions complicated with IAs. In the total of 45 included patients, there were 58 detected IAs. In **Table 3**, we found that 77.8% of patients had a single aneurysm instead of multiple IAs, and 67.2% of aneurysms were narrow-neck instead of wide-neck. This rendered equal priority between clipping surgery and intravascular therapy when considering aneurysm management. For aneurysm location, **Table 3** showed that 32.8% of IAs clustered on ICA C6 segment (ophthalmic segment), where clipping surgery encounters greater difficulties and risks. Hence, intravascular therapy should be privileged here for ophthalmic segment IA complicated with sellar region lesions. Other locations of complicated IAs were C7 (communicating segment, 24.1%), C5 (clinoid segment, 12.1%), PoCA (8.6%), C4 (cavernous segment, 6.9%), MCA (6.9%), C1 (cervical segment, 1.7%), ACA (1.7%), by the sequence of decreased frequency of occurrence. Above all, no matter EEA approach or open cranial approach, total or subtotal tumor excision, treatment strategy of such comorbidity should be comprehensively formulated based on both the features of sellar region lesions and the IAs. It was noted that 4 patients in our study had huge parasellar blood clots far from sellar lesions, indicating hemorrhage instead of tumor apoplexy before the operation. Among these 4 patients, 3 patients chose to deal with sellar lesions first (2 of 3 had subtotal tumor excision), and 1 patient chose conservative treatment without dealing with neither IAs nor sellar lesions.

One of the comforting results of **Table 3** was that 51.7% IAs were micro-aneurysms with a lower risk of rupture, and most patients are in the mild to moderate risk. Given the 0.87% to 1.6% annual rupture rate per IA patient in natural history (9, 24, 25) and aneurysm diameter > 7 mm is an independent risk factor of rupture (25), included patients were divided into three hierarchical subgroups representing different rupture risk. 36 out of 38 (94.7%) patients had IA diameter larger than 3 mm, therefore at middle to low risk. In most cases, it is not recommended to perform aggressive intervention for complicated IAs since they have a relatively low risk of rupture, especially those with a diameter less than 7 mm (9). But the clinical experience on aneurysm size and risk of rupture is established under the premise of the natural history of IAs, it may not work here under the intervention of EEA. Thus, even microaneurysms should arise enough attention during integrated management. Thorough patient-surgeon communication should proceeded before the formulation of an individualized treatment strategy.

In **Tables 3-S1**–**Table 3-S3**, we can find that EEA has been favored by more and more neurosurgeons for tumor resection in the sellar region in recent years due to its specific advantages including minimally invasive, less complication, lower morbidity as well as mortality rates over the conventional craniotomy (26–28). But the incidence of operation-associated massive hemorrhage during EEA could never be ignored. Once IA was found to be complicated with sellar region lesions by preoperative CTA screening, further treatment strategy must be formulated based on the features of IAs (such as number, location, and morphological characteristics) as well as its association with sellar region lesions (such as tumor invasiveness and pointing direction) (29).

The favorable outcome from follow-up highlighted the importance of CTA as a routine preoperative evaluation in the formulation of individualized treatment strategy. CTA is a non-invasive imaging modality with lower cost, faster acquisition, and great accessibility. A meta-analysis conducted by Menke J et al. revealed that the sensitivity and specificity for intracranial aneurysm detection were over 97% (30). Also, relevant yielded outcomes had a 96% sensitivity for IAs with maximal diameters ≥ 3 mm (31).

There are some limitations to our study. First, the sample size of 45 patients is relatively small, though already has been the largest cohort among literatures. Second, we didn’t recruit a healthy population as control. Third, data was manually collected and analyzed so there might exist a certain bias. Fourth, this is a retrospective observational study, the further randomized evaluation may be acquired in a more generalized population. 

## Conclusions

The overall prevalence of sellar region lesions co-existed with the IAs is 11.1%. In the era of EEA for the sellar region lesions complicated with IAs, specific and individualized treatment strategy should be carried out during the clinical management. And we strongly recommend that CTA be considered as routine cerebrovascular screening before EEA to guarantee the safety of procedure.

## Data Availability Statement

The raw data supporting the conclusions of this article will be made available by the authors, without undue reservation.

## Ethics Statement

The studies involving human participants were reviewed and approved by Institutional Review Board and Ethics Committee of West China Hospital, Sichuan University. Written informed consent to participate in this study was provided by the participants’ legal guardian/next of kin. Written informed consent was obtained from the individual(s), and minor(s)’ legal guardian/next of kin, for the publication of any potentially identifiable images or data included in this article.

## Author Contributions

SY and YL are co-first authors and contributed equally to this article. Correspondence addressed to RT (tianrui17419@wchscu.cn) and LZ (zhlxlll@163.com). All authors contributed to the article and approved the submitted version.

## Funding

This research was funded by 1) National Key R&D Program of China, No. 2018YFA0108603; 2) National Key R&D Program of China, No. 2018YFA0108604; 3) 1·3·5 Project for Disciplines of Excellence, West China Hospital, Sichuan University, No. 2021HXFH014; 4) Clinical Research Innovation Project, West China Hospital, Sichuan University, No. 2019HXCX07.

## Conflict of Interest

The authors declare that the research was conducted in the absence of any commercial or financial relationships that could be construed as a potential conflict of interest.

## Publisher’s Note

All claims expressed in this article are solely those of the authors and do not necessarily represent those of their affiliated organizations, or those of the publisher, the editors and the reviewers. Any product that may be evaluated in this article, or claim that may be made by its manufacturer, is not guaranteed or endorsed by the publisher.
