# Supplementary material for: Sellar Region Lesions and Intracranial Aneurysms in the Era of Endoscopic Endonasal Approach
Source: Front Endocrinol (Lausanne). 2021 Jan 4;12:802426. doi: 10.3389/fendo.2021.802426 (PMC8763682; doi:10.3389/fendo.2021.802426)
Supplement: Supplementary file 1 [file DataSheet_1.pdf]

Table 2-S1. The operation information among patients presenting different tumor invasiveness.

**Operation information (n = 40)**	**Invasiveness of sellar region tumors**	
	**Noninvasive**	**Invasive**
Endoscopic or open surgery	31	9
EEA	28	7
Craniotomy	3	2
Excision degree	31	9
Total excision	15	4
Subtotal excision	16	5

Table 2-S2. Preoperative and postoperative hormone loads in different subtypes of sellar region lesions.

**Histological subtype**	**Sample size**	**Mean hormone loads** (min, max[preoperative; postoperative])	**Surgical approaches**
Pituitary adenoma	38		
Corticotroph adenoma	1	94.76;157.6	EEA (1/1)
Somatotroph adenoma	2	25.96(6.61, 45.3);5.11(0.72, 9.49)	EEA (2/2)
Lactotroph adenoma	10	249.85(24.44, 1517);25.63(14.9, 68.15)	EEA (10/10)
Thyrotroph adenoma	1	11.21;-	EEA (0/1)
Gonadotroph adenoma	1	0.11;-	EEA (0/1)

NOTES: The hormone loads referred in the table is adrenocorticotropic hormone for corticotroph adenoma, growth hormone for somatotroph adenoma, prolactin for lactotroph adenoma, thyroid stimulating hormone for thyrotroph adenoma, and testosterone for gonadotroph adenoma (because the only patients with gonadotroph adenoma is a male) respectively. The postoperative hormone loads were examined during the hospitalization. Some patients of lactotroph adenoma, thyrotroph adenoma, and gonadotroph adenoma were lack of postoperative laboratory data.

Abbreviation mentioned in the table: EEA, endoscopic endonasal approach, and the surgical approaches referred in the table including EEA and craniotomy.

Table 3-S1. The surgical methods among patients with different IAs size.

**Operation information (n = 38)**	**Size of the IAs**		
	**Giant**	**Middle**	**Micro**
Endoscopic or open surgery	2	14	22
EEA	2	11	21
Craniotomy	0	3	1
Excision degree	2	14	22
Total excision	1	7	11
Subtotal excision	1	7	11

NOTES: Giant IAs, d⩾10mm; middle IAs, 3mm⩽d<10mm; micro IAs, d<3mm; The patient would be classified into different subgroup based on the size of its maximal IA among multiple IAs population. Patients with IAs without description of size (n=2) were excluded from the statistical analysis.

Table 3-S2. The surgical methods among patients with different dome-to-neck ratio of IAs.

**Operation information (n = 38)**	**Dome-to-neck ratio of the IAs**	
	**Wide-neck**	**Narrow-neck**
Endoscopic or open surgery	9	29
EEA	7	27
Craniotomy	2	2
Excision degree	9	29
Total excision	6	13
Subtotal excision	3	16

NOTES: The patients with multiple IAs, among which at least one of them is classified as wide-neck IA, he/she will be classified into the wide-neck subgroup, the narrow-neck subgroup otherwise. Patients with IAs without description of dome-to-neck ratio (n=2) were excluded from the statistical analysis.

Table 3-S3. The surgical methods among patients with different direction of IAs.

**Operation information**	**Direction of the IAs**		
**(n = 38)**	**Point to**	**In parallel with**	**Deviate from**
Endoscopic or open surgery	19	6	13
EEA	18	6	11
Craniotomy	1	0	2
Excision degree	19	6	13
Total excision	8	3	8
Subtotal excision	11	3	5

NOTES: The patients with multiple IAs, among which at least one of them is recognized as pointing to the cavernous sinus, he/she will be classified into the "point to" subgroup; at least one of them is recognized as in parallel with the cavernous sinus, he/she will be classified into the "in parallel with" group. Patients with IAs without description of its direction (n=2) were excluded from the statistical analysis.
